# Clinical Proteomics Identifies Urinary CD14 as a Potential Biomarker for Diagnosis of Stable Coronary Artery Disease

**DOI:** 10.1371/journal.pone.0117169

**Published:** 2015-02-10

**Authors:** Min-Yi Lee, Chun-Hao Huang, Chao-Jen Kuo, Chen-Lung Steve Lin, Wen-Ter Lai, Shyh-Horng Chiou

**Affiliations:** 1 Graduate Institute of Medicine, College of Medicine, Kaohsiung Medical University, Kaohsiung, Taiwan; 2 Division of Cardiology, Department of Internal Medicine, Kaohsiung Municipal Ming-Sheng Hospital, Kaohsiung, Taiwan; 3 Quantitative Proteomics Center, Center for Research Resources and Development, Kaohsiung Medical University, Kaohsiung, Taiwan; 4 Department of Surgery, Kaohsiung Medical University Hospital, Kaohsiung, Taiwan; 5 Division of Cardiology, Department of Internal Medicine, Kaohsiung Medical University Hospital, Kaohsiung, Taiwan; 6 Institute of Biological Chemistry, Academia Sinica, Taipei, Taiwan; University of Milan, ITALY

## Abstract

Inflammation plays a key role in coronary artery disease (CAD) and other manifestations of atherosclerosis. Recently, urinary proteins were found to be useful markers for reflecting inflammation status of different organs. To identify potential biomarker for diagnosis of CAD, we performed one-dimensional SDS-gel electrophoresis followed by liquid chromatography coupled with tandem mass spectrometry (LC-MS/MS). Among the proteins differentially expressed in urine samples, monocyte antigen CD14 was found to be consistently expressed in higher amounts in the CAD patients as compared to normal controls. Using enzyme-linked immunosorbent assays to analyze the concentrations of CD14 in urine and serum, we confirmed that urinary CD14 levels were significantly higher in patients (n = 73) with multi-vessel and single vessel CAD than in normal control (n = 35) (*P* < 0.001). Logistic regression analysis further showed that urinary CD14 concentration level is associated with severity or number of diseased vessels and SYNTAX score after adjustment for potential confounders. Concomitantly, the proportion of CD14+ monocytes was significantly increased in CAD patients (59.7 ± 3.6%) as compared with healthy controls (14.9 ± 2.1%) (P < 0.001), implicating that a high level of urinary CD14 may be potentially involved in mechanism(s) leading to CAD pathogenesis. By performing shotgun proteomics, we further revealed that CD14-associated inflammatory response networks may play an essential role in CAD. In conclusion, the current study has demonstrated that release of CD14 in urine coupled with more CD14+ monocytes in CAD patients is significantly correlated with severity of CAD, pointing to the potential application of urinary CD14 as a novel noninvasive biomarker for large-scale diagnostic screening of susceptible CAD patients.

## Introduction

Coronary artery disease (CAD) is the most common type of heart disease and cause of heart attacks [1]. Recent research has shown that inflammation plays a key role in CAD and other manifestations of atherosclerosis [1]. Cytokine or growth factors produced in the inflamed intima induces monocytes entering the plaque to differentiate into macrophages, leading to the development of atherosclerosis [2]. Effector molecules from immune cells that dominate early stage of atherosclerotic lesions accelerate progression of the lesions and further elicit acute coronary syndromes. These inflammatory factors, such as C-reactive protein (CRP) or monocyte chemoattractant protein-1 (MCP-1), represent attractive biomarkers for the prediction of risk for developing CAD [3]. Although these biomarkers hold promises, the invasive procedures for their clinical applications still emphasize an urgent need for non-invasive biomarkers that can correlate with severity of CAD.

Urine has especially become one of the most attractive body fluids in biomarker discovery as it can be obtained non-invasively in large quantities and is stable as compared with other body fluids. Recently, urinary proteins were also found to be useful markers for reflecting inflammation status of different organs. However, applying approaches for biomarker discovery to urinary samples have been encumbered by concerns for reproducibility and poor standardization of protocols until lately the development of state-of-the-art proteomic methodology [4–6].

Proteomic approaches in healthy and pathological samples are especially helpful to facilitate discerning differential protein expression patterns associated with normal and diseased states. Mainly attributable to the advent of emerging proteomics, the analysis and identification of complex protein mixtures in biological tissues have recently become amendable to routine analysis [7–9]. The study of proteins at the level of cellular systems derived from various tissues by proteomics methods has provided a firm basis for understanding the complex proteome profiles of total protein mixtures from complex whole tissues or cells at various disease stages [10]. As a result, proteomics has successfully been used to examine dynamic changes in protein expression of various tissues or body fluids, resulting in the identification of clinically useful biomarkers for disease diagnosis and prognosis [11–13]. Therefore, comparative proteomics analysis of normal and urinary samples from patients with a defined disease stage is particularly valuable for studying the differences of proteins expressions between diseased and normal subjects [7].

In this study, we employ an optimized gel-based coupled with gel-free shotgun proteomics strategy for urine biomarker discovery and a mechanistic investigation. Among the proteins differentially expressed in urine samples, monocyte antigen CD14 was found to be consistently expressed in higher amounts in the CAD patients as compared to normal controls. We have further made an endeavor to examine the differential manifestations of CD14 by proteomics and enzyme-linked immunosorbent assays in urine and flow cytometry on monocytes of normal control and CAD patients to provide some insight into the functional role of CD14 underlining the diagnostic or prognostic value for large-scale screening of CAD patients.

## Materials and Methods

### Study population

Seventy-three patients with angiographically proven CAD and 35 subjects with normal coronary arteries (mean age 65.5 ± 12.5 years), were enrolled in this successive study from 1 January to 30 June, 2012. The study was approved by the medical ethics committee of Kaohsiung Municipal Hospital and written informed consents were obtained from all participants. None of the patients had a history of active infectious disease, urinary tract infection, prior stroke, acute coronary syndrome or malignancies. Smoking status was recorded as three types, i.e. current smokers, ex-smokers or non-smokers. Body mass index (BMI) was calculated as weight (in kilograms) divided by height squared (in meters). A venous blood sample was collected for each patient on the day of examination after overnight fasting for at least 8 h. The following parameters were obtained by standard biochemical analysis techniques, including total cholesterol, low-density lipoprotein cholesterol, high-density lipoprotein cholesterol, triglycerides, serum creatinine, high sensitivity C-reactive protein (hs-CRP) and fibrinogen. Urine and plasma samples were collected before coronary angiography and stored at −80°C until analysis.

### Measurements of urinary protein, microalbumin and creatinine

Urinary protein and microalbumin were measured using commercial kits according to the manufacturer’s instructions (BioSystems, Barcelona, Spain). Serum and urinary creatinine levels were analyzed by a UV-Visible spectrophotometer at 505 nm by a standard spectrophotometric protocol. The ratio of urinary microalbumin to creatinine (UACR) was then calculated.

### Urine proteomics based on one-dimensional (1-D) gel

Specimens of the first morning urine of about 10 ml each (collected between 8 and 10 A.M.) were collected in a sterile container for every test subject. This urine collection protocol is in accord with the “standard protocol for urine collection” currently under development by the Human Urine and Kidney Proteome Project and European Kidney and Urine Proteomics Action (EuroKUP) networks (www.eurokup.org; www.hukpp.org). The specimens were subsequently centrifuged at 1000 x g for 5 min to remove cellular debris. Precipitation of proteins was carried out in 10% trichloroacetic acid (TCA) containing 6 mM dithiothreitol (DTT) on ice for 30 min, and protein harvested by centrifugation at 14,000 rpm (18,078 x g) [14]. The washed protein pellet was washed twice with ice-cold 100% acetone/6 mM DTT. The pellet samples were always stored at −80°C until further analysis. For analysis, pellets were re-suspended in 0.4 ml SDS-containing loading buffer (10% SDS, 0.3125 M Tris-HCl, pH 6.8, 10% glycerol, 0.5 M DTT, 0.01% bromphenol blue) heating for 5 min at 95°C. About 10 μg of proteins (estimated based on a BCA protein-assay reagent kit (Pierce, Rockford, IL)) were loaded on 12.5% 1-D SDS–PAGE for protein separation with a 5% stacking gel. The gels were stained with Coomassie brilliant blue R-250 and destained in 10% methanol/ 7% acetic acid.

### In-gel digestion and nanoLC-ESI-MS/MS

Based on the 1-D SDS-gel analysis of samples collected from 8 patients with different severity of CAD, the protein bands separated on the gels were sliced and destained three times with 25 mM ammonium bicarbonate buffer (pH 8.0) in 50% acetonitrile (ACN) for 1 h. The gel pieces were dehydrated in 100% ACN for 5 min and then dried for 30 min in a vacuum centrifuge. Enzyme digestion with trypsin and the peptide fragments analyzed by nanoLC-ESI MS/MS were essentially according to the previous report [14]. Proteins were identified in the NCBI databases based on MS/MS ion search with the search program MASCOT as described [14, 15].

### Western blot analysis

After gel transfer, the membranes were soaked in Tris-buffered saline containing 5% nonfat dry milk powders and 0.1% Tween-20 (TBS-T) for one hour, followed by incubation with rabbit polyclonal antibody against CD14 (anti-CD14) (GTX101342; Genetex, San Antonio, TX, USA) overnight at 4°C. After three washes with TBS-T, the membranes were then incubated with a solution of anti-rabbit Ig conjugated with horseradish peroxidase. After 1-h incubation at room temperature, the membranes were washed three times with TBS-T and the membrane blots were developed by using enhanced chemiluminescence substrate (ECL kit; Pierce, Rockford, IL, USA).

### Measurement of soluble CD14 levels in serum and urine

Serum and urinary CD14 levels were measured by human CD14 enzyme linked immunosorbent assay (ELISA) kit (HK320; Hycult Biotechnology, Uden, Netherlands). Serum and urine samples were diluted and assayed according to the manufacturer’s instructions. Intra-assay variability was determined by evaluating 5 serum and 5 urinary samples under the same assay conditions, showing that a coefficient of variation (CV) between 5% and 9%. Inter-assay variability was determined by measuring 5 serum and 5 urinary samples in 5 consecutive assay runs, which showed a CV between 7% and 11%. The coefficients of variation for the intraassay and interassay of this AGT ELISA were found to be 4.8% and 5.3%, respectively. The detection limit for CD14 was 10 pg/mL.

### Flow cytometry of CD14+ monocytes

We investigated CD14+ monocytes in 5 patients with angiographically proved CAD and 5 age- and sex- matched control subjects using flow cytometry. All of the patients had no prior history of active infectious disease, urinary tract infection, recent stroke or acute myocardial infarction in the past 6 months.

The surface expression of CD14 on peripheral blood mononuclear cells (PBMCs) was analyzed on the day of collection by direct staining with fluorescein isothiocyanate (FITC)-conjugated mouse anti-CD14 monoclonal antibody (clone 61D3; eBioscience, San Diego, CA, USA). PBMC were incubated with saturating concentrations of FITC-labeled anti-CD14 mAb for 30 min at 4°C in the dark. PBMCs were washed well with PBS and re-suspended in PBS with 1% FCS. Cells were analyzed by using a LSRII flow cytometer (Becton Dickinson, Heidelberg, Germany) and data were analyzed by FCS Express v3 Software System (Becton Dickinson, Heidelberg, Germany). The monocytes were specifically analyzed by selective gating based on parameters of forward and side light scatters. The absolute count of CD14+ monocytes was calculated from the total leukocyte count and the proportion of monocytes in the total leukocytes, as determined by light scattering.

### Quantitative real time polymerase chain reaction (qRT-PCR)

qRT-PCR was employed to assay the CD14 levesl of patients with and without CAD. PBMCs were isolated by centrifugation. The PRISM 7900 Sequence Detection System (Applied Biosystems, Foster City, CA, USA) was used for real-time PCR analysis. Specific primers were designed using Primer Express software (Applied Biosystems, Foster City, CA, USA) using sequences accessed through Gen-Bank and checked for specificity using Blast-search. CD14 sequence was used to design the qRT-PCR primer as follows: CD14 5’-TGCGACCACGCCAGAAC-3’ (sense) and 5’-GCAGACGCAGCGGAAATC-3’ (antisense). GAPDH (endogenous control) sequence was used to design primers for qRT-PCR as follows: 5’-GGTGGTCTCCTCTGACTTCAACA-3’ (sense) and 5’-GTTGCTGTAGCCAAATTCGTTGT-3’ (antisense). Real-time polymerase chain reaction (PCR) was performed using the 1× SYBR Premix EX Taq (Perfect Real Time) premix reagent (Takara, Japan), and added 500 ng cDNA to a final volume of 10 μl. Samples were run in triplicate. Real-time PCR performed on ABI PRISM 7900 Sequence Detection System (Applied Biosystems, Foster City, CA, USA). Fluorescence was measured after each of the repetitive cycles. For each gene, cycle threshold values were determined from the linear region of the amplification plot. Expression levels of mRNA were normalized relative to GAPDH mRNA levels. A melting point dissociation curve generated by the instrument was used to confirm that only a single product was present. Each PCR performed also included triplicate wells containing no template as controls in which nuclease-free water was added to reaction wells.

### Coronary angiographic evaluation

Coronary angiography was performed for all subjects by the Judkin’s method. Two different scores were used to evaluate the angiographic severity and extent of CAD: the number of stenosed (> 50% reduction of luminal diameter) or occluded vessels (one- to three-vessel disorder states), and the complexity and severity scoring method according to the SYNTAX score [16]. Scoring of all coronary angiograms was done visually by one experienced observer who was blinded to the existing clinical and laboratory data. The intra-class correlation coefficient for intra-rater reliability was 1.0 (one to three vessel disorder score), and 0.93 (tertiles of the SYNTAX score). Multi-vessel CAD was defined as patients with two or more stenosed vessels or significant lesions at left main coronary artery.

### Statistical analysis

Baseline characteristics were calculated for patients and controls. Numeric data are presented as means ± standard deviation (SD) when they are normal distributed, otherwise presented as median and interquartile range, and categorical data are presented as frequencies and percentages. Normal Quantile plot (Q-Q plot) indicated the log of urinary CD14 values was approximately normally distributed. Comparisons between three groups were analyzed by one-way ANOVA using a two-tailed test for variables with a Gaussian distribution. Comparisons between ratios were carried out using the χ2 test. Furthermore, logistic regression analysis was performed to assess clinical determinants between independent variables and presence of CAD. We divided urinary CD14 levels into quartiles based on the distribution among all subjects. To evaluate the relationship between urinary CD14 levels and other risk factors, we calculated mean levels of risk factors per urinary CD14 quartile. Interquartile comparison was performed with the mean or median of each urinary CD14 quartile. To estimate the relative risk of prevalent CAD, we calculated odds ratios (OR) and corresponding 95% confidence intervals (95% CI). ORs were calculated using conditional logistic regression, taking into account the matching for sex and age, and was adjusted for smoking status, diabetes, body mass index (BMI), low density lipoprotein (LDL) cholesterol, high density lipoprotein (HDL) cholesterol, systolic blood pressure and high sensitivity C-reactive protein (hs-CRP) levels. Data analysis was performed with SPSS software (version 15; SPSS Inc., Chicago, IL, USA). A probability value *P*< 0.05 was considered to show statistical significance in the analyzed sets of data.

### Quantitative shotgun proteomics

The methods for shotgun proteomics on urine samples were described separately in S1 Materials and Methods.

## Results

### Proteomic analysis differentiates urine of normal controls from CAD patients

**Figure 1:**
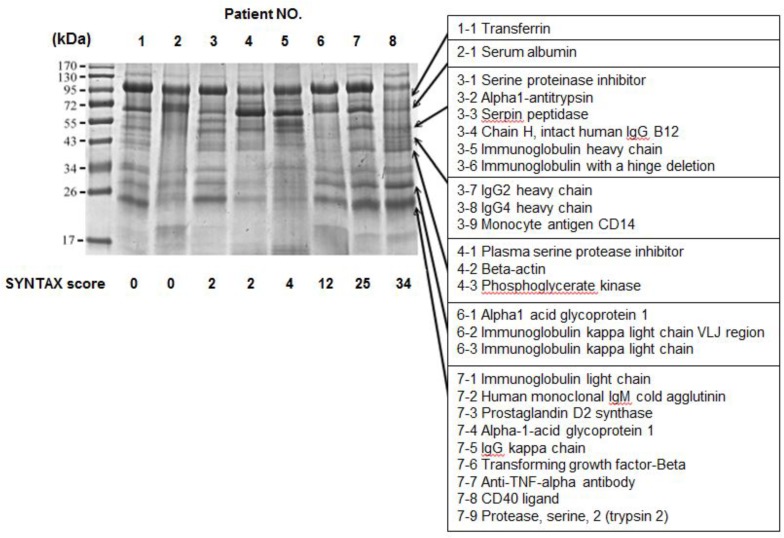
1-D gel analysis of urinary proteins from healthy subjects (patient NO. 1 and 2) and CAD patients (patient NO. 3 to 8). A total of 10 μg of urinary proteins derived from healthy subjects and patients with different severity of CAD manifested with SYNTAX scores were used for 1-D gel comparative analysis of urinary proteins. Proteomic bands shown with different intensities were excised, trypsin-digested and followed by injection to LC-MS/MS. The identified proteins/peptides were shown at the right panel, related to Table 1.

**Figure 2:**
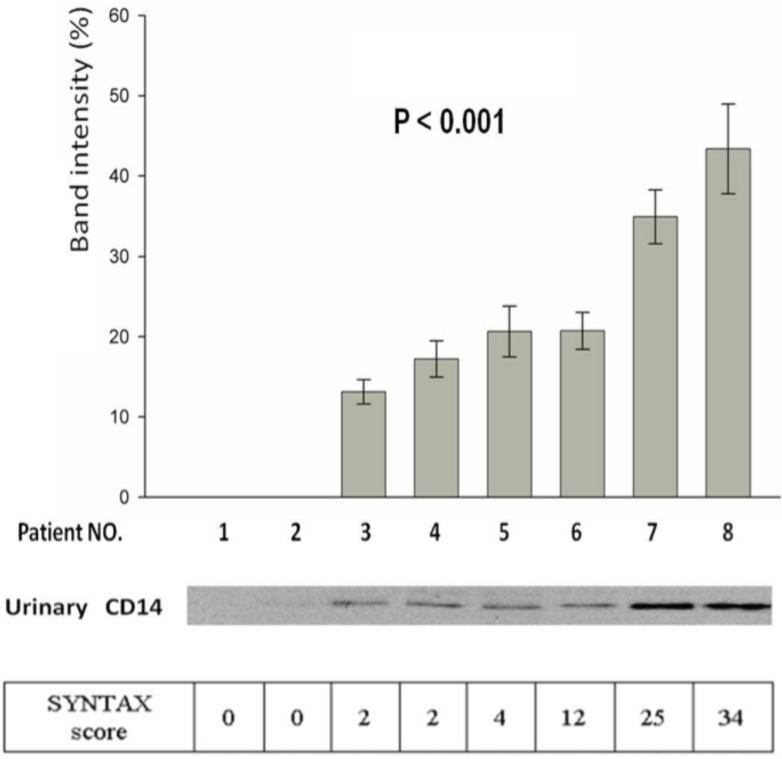
Western blot analysis of urinary CD14. Equal amounts of total protein (10μg) isolated from the urine of healthy subjects and CAD patients were loaded onto each lane. The CD14 bands (at approximately 48 kDa) were clearly detected in all urine samples of patients with CAD, whereas it was undetectable in healthy subjects. The band intensity was analyzed by scanning densitometry software (Multi Gauge V3.0).

To identify potential urinary markers for CAD patients, 1-D SDS– PAGE gel is an ideal method to compare the protein differentially expressed in urine. After protein extraction of urine samples from incremental CAD severity of 8 subjects (SYNTAX score from 0 to 34), 10 μg protein from each sample was subjected to SDS-PAGE analysis (Fig. 1). The major bands with intensity difference were excised from the gels and analyzed by LC-MS/MS, leading to identification of 26 proteins and peptides (Table 1). 4 top candidates, monocyte antigen CD14, prostaglandin D2 synthase, transforming growth factor-beta and CD40 ligands, were further validated using Western blot analysis (data not shown). However, only CD14, a 55 kDa glycoprotein, showed markedly increased intensities (*P* < 0.001) from the urine samples of CAD patients while the CD14 protein band was barely detectable in the urine of healthy controls (Fig. 2). These data illustrate the facile application of using proteomic approaches to identify differentially expressed proteins in urine, resulting in the identification of urinary CD14 as a potential biomarker for CAD patients.

**Table 1:** Summary of excreted proteins in the urine of CAD patients.

Spot no.^a^	Function	Protein name	GenInfo ID^b^	Accession no.	Peptide queries matched	Cov (%)	pI^c^	M.W.^d^	Score
1–1	Energy metabolism	Transferrin	gi|37747855	AAH59367	27	32%	6.81	77030	529
2–1	Transport	Serum albumin	gi|28592	CAA23754	63	50%	6.05	69321	1093
3–1	Serine protease inhibitor	Serine proteinase inhibitor	gi|50363217	NP_000286	43	56%	5.37	46707	817
3–2	Serine protease inhibitor	Alpha1-Antitrypsin	gi|157831596	1KCT_A	43	60%	5.37	44223	817
3–3	Serine protease inhibitor	Serpin peptidase inhibitor	gi|15080499	AAH11991	41	56%	5.36	46693	804
3–4	Humoral immune system	Chain H, Intact Human IgG B12	gi|15825647	1HZH_H	16	19%	8.59	50385	222
3–5	Antigen binding	Immunoglobulin heavy chain	gi|272982598	ACZ97422	15	16%	8.13	51775	204
3–6	Antigen binding	Immunoglobulin With A Hinge Deletion	gi|494350	1MCO_H	13	17%	9.04	46823	191
3–7	Antigen binding	IgG2 heavy chain	gi|14030849	AAG00910	10	14%	7.94	49488	150
3–8	Antigen binding	IgG4 heavy chain	gi|9857759	AAG00912	7	19%	5.72	42895	130
3–9	Inflammatory response	Monocyte antigen CD14	gi|3983127	AAC83816	6	18%	5.85	40065	136
4–1	Serine protease inhibitor	Plasma serine protease inhibitor	gi|180550	AAA35688	6	14%	9.38	45772	347
4–2	Structural constituent of cytoskeleton	Beta-actin	gi|14250401	AAH08633	9	25%	5.56	40978	314
4–3	Phosphoglycerate kinase activity	Phosphoglycerate kinase	gi|4505763	NP_000282	3	10%	8.3	44586	121
6–1	Regulation of immune system process	Alpha-1-acid glycoprotein 1	gi|112877	P02763	14	7%	4.93	23497	252
6–2	Immune response	Immunoglobulin kappa light chain VLJ region	gi|21669329	BAC01689	9	24%	6.73	28497	234
6–3	Immune response	Immunoglobulin kappa light chain	gi|110626504	ABG79005	9	30%	7.85	23552	234
7–1	Antigen binding	Immunoglobulin light chain	gi|149673887	BAF64541	20	47%	6.97	23380	684
7–2	Immune response	Human monoclonal IgM Cold agglutinin	gi|10835792	1QLR_A	17	51%	5.75	23266	584
7–3	Prostaglandin-D synthase activity	Prostaglandin D2 synthase	gi|54696702	AAV38723	7	21%	7.66	21015	305
7–4	Regulation of immune system process	Alpha-1-acid glycoprotein 1	gi|112877	P02763	5	7%	4.93	23497	133
7–5	Antigen binding	IgG kappa chain	gi|4176418	BAA37169	11	47%	6.92	23404	580
7–6	Growth factor activity	Transforming growth factor-Beta	gi|215794746	3EO0_A	11	47%	6.19	23390	561
7–7	Anti tumor necrosis factor	Anti-TNF-alpha antibody	gi|11275306	BAB18253	11	48%	7.75	23365	456
7–8	Inflammatory response	CD40 ligand	gi|20150126	1I9R_L	14	39%	6.48	23843	335
7–9	Serine-type endopeptidase activity	Protease, serine, 2 (trypsin 2)	gi|74353564	AAI03998	1	4%	4.78	26455	48

^a^ The number correspond to those showed in Fig. 1.

^b^ According to the NCBI database.

^c^ Theoretical pI.

^d^ Theoretical molecular mass.

### Urinary but not serum CD14 predicts the severity of CAD

**Figure 3:**
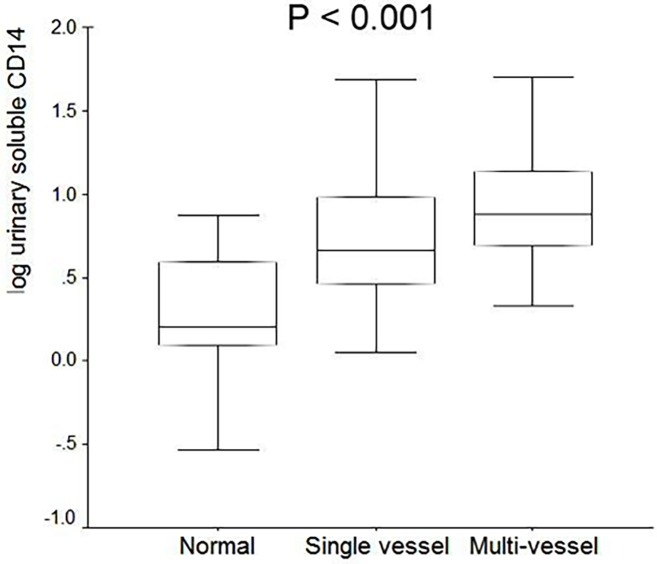
ELISA measurements of urinary and serum CD14 among subjects with different severity of coronary artery disease. There were significant differences in urinary CD14 levels in patients with different severity of CAD.

To confirm urinary CD14 being a verifiable biomarker, we further perform ELISA to screen clinical samples in a large-scale study of patients with differential severities of CAD. S1 Table shows the baseline biochemical data regarding CAD patients and control subjects. The prevalence of traditional risk factors such as age, gender, history of diabetes mellitus and serum creatinine was higher in patients with single- or multi-vessel CAD than in controls (*P* < 0.05). White cell counts, hs-CRP, fibrinogen levels, urinary microalbumin and UACR were not significantly different among CAD patients and controls. However, urinary CD14 levels measured by ELISA in patients with single or multi-vessel CAD were significantly increased in comparison with patients with normal coronary angiography (Fig. 3). In contrast, there is no significant difference of serum CD14 levels among these three groups (S1 Table). Taken together, these results indicate that the level of urinary CD14 reflects the severity of CAD at different stages, supporting its application as a biomarker.

### ROC curve analysis assesses urinary CD14 as a biomarker for CAD

**Figure 4:**
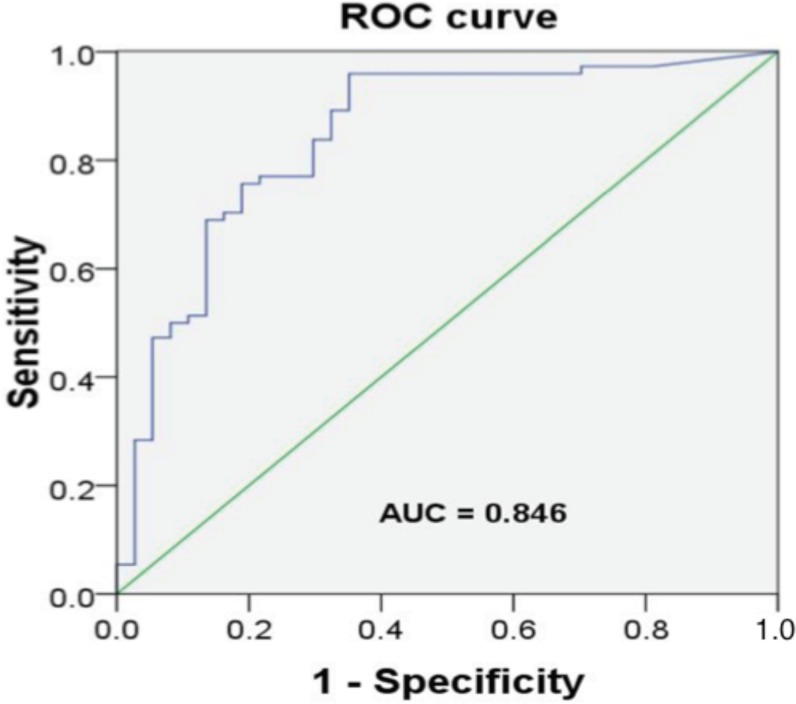
Receiver-operating characteristic (ROC) plots of urinary CD14 for diagnosis of coronary artery disease. Using a threshold value of 3.51, urinary CD14 has a sensitivity of 0.838 and a specificity of 0.703, a fairly good and significant biomarker for the prediction of CAD.

As shown in S2 Table, The association of urinary CD14 quartiles with CAD was not attenuated after adjusting for CVD risk factors. Only age and SYNTAX score were associated positively with urinary CD14 levels. In logistic regression, quartiles of urinary CD14 levels were positively associated with SYNTAX score when compared with normal controls following adjustment for age, gender, medications, and all traditional CVD risk factors, and even with further adjustment for serum creatinine and hs-CRP levels (S3 Table). The urinary CD14 levels above the median were strongly associated with patients of CAD in fully adjusted models that included traditional CVD risk factors, serum creatinine, and hs-CRP (odds ratio 3.336 95% CI: 1.232 to 9.032; *P* = 0.018). Using a threshold value of 3.51 μg/mL, urinary CD14 has a sensitivity of 0.838 and a specificity of 0.703 for the prediction of CAD, implicating CD14 exhibiting as a fairly exceptional and significant biomarker for CAD (Fig. 4).

### Proportion of CD14+ monocyte is higher in CAD patients than in normal controls

**Figure 5:**
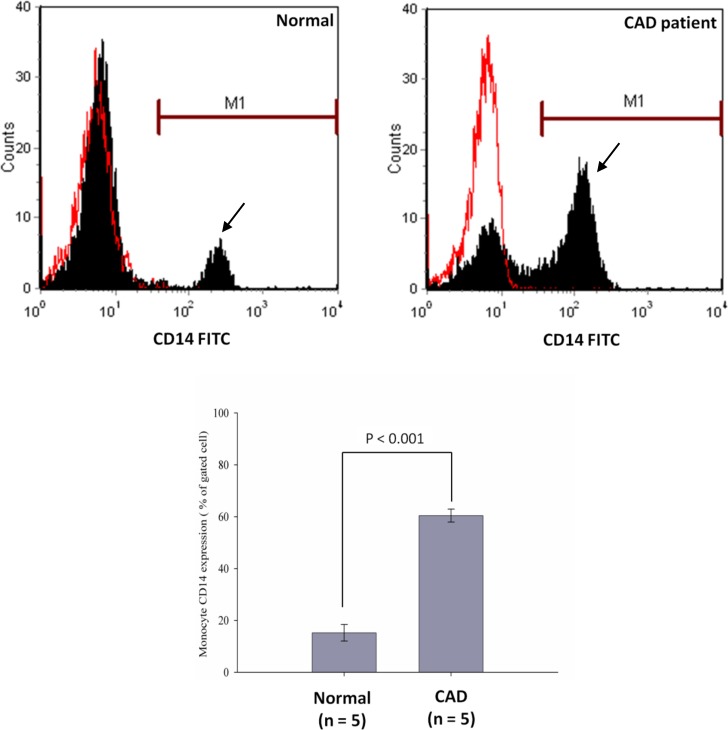
Expressions of CD14+ monocytes in healthy subjects and CAD patients. CD14 surface expressions were measured by flow cytometry as described in the Methods section. The numbers of CD14+ monocytes were determined before carrying out coronary angiography on patients. There was a significant difference in CD14+ monocyte counts between healthy subjects and CAD patients. Arrows indicate monocyte fractions which were estimated by the gating procedure according to forward scatter/sideward scatter (FSC/SSC) dot plot.

**Figure 6:**
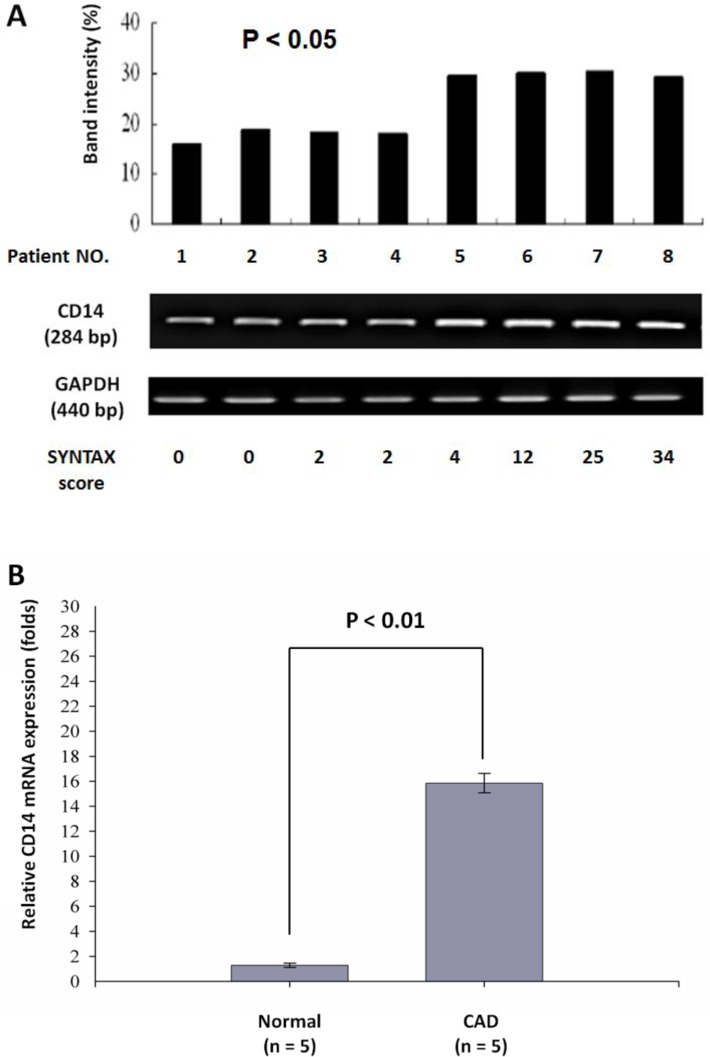
Induction of CD14 gene expression in peripheral blood mononuclear cells from healthy subjects and CAD patients. (**A**) RT-PCR (reverse transcription-PCR) amplification of total RNA from CAD patients reveals CD14 mRNA product (284 bps) corresponding to the whole CD14 gene (356 bps) in genomic DNA. Amplified product of GAPDH was utilized as a reference mRNA marker. (**B**) Quantitative real-time PCR (qRT-PCR) was performed using primers specific for CD14 and GAPDH. Transcript levels were normalized to that of GAPDH mRNA. The CD14 mRNA expression is shown as the folds of transcript levels in CAD patients relative to healthy subjects.

To investigate the potential mechanism leading to higher level of urinary CD14 in CAD patients, we hypothesize that it may be related to the number of CD14+ monocytes. To this end, we determined the proportions of CD14+ monocytes in peripheral blood samples from 5 CAD patients and 5 age-matched healthy controls using flow cytometry. Although we performed the flow cytometry study only in 5 patients and 5 controls, their clinical and biochemical characteristics were well controlled between these two groups (S4 Table). Hence, the expression of CD14+ monocytes can be distinguished between patient and control groups. Fig. 5 showed representative staining patterns of surface expression of membrane-bound CD14 on monocytes from a patient with CAD and a healthy individual. The proportion of CD14+ monocytes was significantly increased in CAD patients (59.7 ± 3.6%) as compared with healthy controls (14.9 ± 2.1%) (*P* < 0.001). To further confirm this result, we isolated peripheral blood mononuclear cells and examined their CD14 mRNA expression level by semi-quantitative RT-PCR. As shown in Fig. 6A, CD14 mRNA was found to be up-regulated in CAD patients. Moreover, to accurately quantify the levels of CD14 mRNA expression, 10 samples consisting of 5 controls and 5 CAD patients were further analyzed by real-time PCR. The amount of CD14 mRNA was found to be more than 15-fold higher in CAD patients than in controls. Statistical analysis revealed that this up-regulated CD14 mRNA expression observed in CAD patients was significant (*P* < 0.01) (Fig. 6B). Of note, although we found the proportion of CD14+ monocytes was significantly increased in CAD patients as compared with healthy controls, we did not observe significant correlation between CAD severity and the number of CD+14 monocytes or CD14 mRNA in the patients. Together, these results indicate that CAD patients tend to have more CD14+ monocytes than normal controls, which suggests that the prospective mechanism for the increased release of urinary CD14 in CAD patients may be attributable to the up-regulation of CD14 mRNA expression resulting in higher expression of membrane-bound CD14 antigens on monocytes from CAD patients as compared to healthy controls.

### Shotgun proteomics reveals CD14-associated inflammatory response networks in CAD

**Figure 7:**
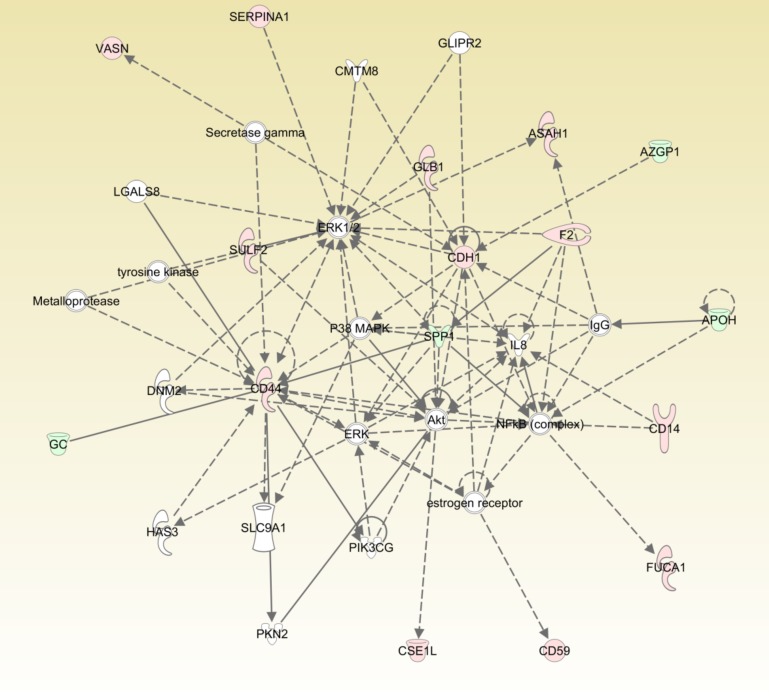
Schematic representation of derived pathways associated with CD14 involved in related interaction. The networks of these identified proteins mapping to the canonical pathways from Ingenuity Pathways Analysis (IPA, Ingenuity Systems) library were employed for the analysis of protein molecules with up- and down-regulation between normal and diseased patients. These identified up- and down-regulated proteins displayed in red and green, respectively and with different shapes to indicate different functions were classified to build the derived signaling networks. The gray arrows with solid and dashed lines indicate the direct and indirect interrelationships, respectively between molecules. Proteins, which were not in our identified category but present in canonical pathway database based on their functional annotation, were displayed in white color and recruited to establish the possible molecular interaction. All drawn arrows were cited and supported by at least one reference from the literature, textbooks, or canonical information stored in the Ingenuity Knowledge Base.

In order to systematically map the protein networks related to urinary CD14 and identify potential mechanisms, we performed shotgun proteomics coupled with stable isotope labeling for quantitative analysis. As shown in S5 Table, more than 100 identified proteins in the MS/MS database with differential expression based on stable isotope (deuterium/hydrogen, D/H) labeling. Similar to previous result of 1-D gel proteomic analysis, CD14 had dramatically higher level in the mixed urine of five CAD patients as compared to five normal controls based on the obtained D/H quantification ratio of 9.682 (S5 Table). In addition, these identified proteins showing up- and down-regulation between normal and diseased patients were further mapped to the canonical pathways from Ingenuity Pathways Analysis library (Fig. 7). Interestingly, several important inflammation regulators, such as nuclear factor kB (NF-κB) and IL-8, are involved in CD14-related inflammatory response networks. These results attest to the observations that the high level of urinary CD14 or the increased number of CD14+ monocytes may result from the chronic inflammation in CAD patients.

## Discussion

Inflammation is contended to play a major role in the pathogenesis of atherosclerotic CAD. Atherosclerosis, the main cause of CAD, is now considered as an inflammatory disease in which immune mechanisms interact with metabolic risk factors to initiate, propagate, and activate lesions in the physiological artery system [1]. Atherosclerosis and its clinical complications are thus asserted to be an inflammatory syndrome in which an ongoing systemic inflammatory response is combined with the accumulation of immune myeloid cells in the atherosclerotic plaque. Above all, monocytes and macrophages play key roles in development and progression of the atherosclerotic plaque. Macrophages can differentiate into foam cells, which are the predominant cell population in the early stage of atherosclerosis [17–19]. In this study we demonstrated that CAD patients had significantly higher levels of urinary CD14 in spite of the fact that the levels of serum CD14 were found not to be different between CAD patients and healthy controls, which corroborated the previous study [20] in ruling out an independent relationship between CD14 genotypes or plasma levels of CD14 and risk of stable CAD in the studied cohort of population. In addition, we also established that urinary and not the plasma CD14 correlates with the severity of CAD, pointing to the potential significance of the expression and release of urinary soluble CD14 in relation to CAD.

Chronic inflammatory state induced by various infections within the vascular wall of animal models has long been suspected to be related to atherosclerosis, prompting researchers to make an extensive search for specific microbial products that may mediate arterial inflammation. Among them, bacterial endotoxin (e.g. lipopolysaccharide [LPS]) [21, 22] appears to play an important role in this aspect of endotoxin-induced pathological alteration in artery vascular system. In gram-negative bacterial infections, bacteria-derived LPS may complex with the serum derived LPS-binding protein. LPS-binding protein could then act as a lipid transfer protein that facilitates the binding of LPS monomers to cellular CD14 [23, 24]. CD14 appears thus to represent the principal binding element of the LPS receptor complex. After LPS binding to leukocyte CD14, there have been shown a cascade of an increase in intracellular Ca^2+^, cellular tyrosine kinase phosphorylation, nuclear factor kB (NF-κB) activation, and cytokine plus chemokine production [25]. Both arms of the immune system, i.e. innate and adaptive immunity, have been implicated in contributing to essentially various stages of atherosclerosis, starting from stages of initiation and progression, and ultimately to the final stage of atherothrombotic complications. Physiological responsiveness to lipopolysaccharide may therefore be correlated with the risk of atherosclerotic disease. The molecular basis of this connection appears to lie in Toll-like receptors that are expressed on cells of the innate immune system. Toll-like receptors are found to bind to lipopolysaccharide, which then govern the strength of antibacterial immune responses exerted by the host [26]. Disparities and differences in the functions of various Toll-like receptors and their signaling pathways are now believed to play a critical role in determining the risk of atherosclerosis [27–29].

Several clinical studies have reported significantly elevated serum levels of CD14 under inflammatory conditions, such as Kawasaki disease [30], atopic dermatitis [31], rheumatoid arthritis [32, 33], and systemic lupus erythematosus [34]. However, the plasma levels of serum CD14 were not found to be different between CAD patients and controls according to the previous report [20]. This finding was in accordance with our results that showed no difference in serum CD14 levels between CAD patients and control subjects (Fig. 3). Several previous studies showed that increased numbers of CD14+CD16+ monocytes are associated with CAD [35]. Our flow cytometric analysis also corroborated that CD14+ monocytes was significantly elevated in CAD patients as compared with healthy controls (Fig. 5). In addition to its function in lipopolysaccharide (LPS) signaling, CD14 may play a role in inflammatory diseases by regulating the innate immune system in response to endotoxin. A previous report [36] provided evidence that CD14 is shed from the cell surface of monocytes rather than internalized. The authors suggested that CD14 shedding induced by monocyte stimulation might play an important role in the regulation of surface CD14 expression. Nevertheless protease-mediated shedding of the mCD14 from leukocytes [37] or direct secretion by monocytes are not the only sources of CD14. CD14 has also be shown to be directly secreted by hepatocytes [38]. Although the relative contribution of CD14 in various cell sources has not been determined, the hepatocytes are probably an important source of circulating CD14. The concentrations of CD14 found in normal human plasma exceed by 1 or 2 logs that of the cell membrane-bound receptor [38]. Therefore we measured both the concentrations of serum CD14 and urinary CD14 in this study. It is of interest to find that there is a close association between urinary concentration of CD14 and the severity of CAD. To date this is the first report on the significant association between urinary CD14 and the risk and disease staging of prospective CAD patients. Further study along the line to look for disease mechanism underlying the correlation of CD14 in urine with the development and progression of CAD is currently in progress.

To date there are several biomarkers which have been used as relevant tools for differentiating patients with and without CAD. These biomarkers include lipocalin-type prostaglandin D synthase [39], electronegative low-density lipoprotein [40], oxidized-LDL/beta(2)-glycoprotein I complexes [41], monocyte chemo-attractant protein-1 [42], eosinophil cationic protein [43], high sensitive cardiac troponin T [44], and homocysteine [45]. All of these biomarkers are obtained and assessed on the day of patient enrollment in plasma or serum that may be interfered by a lot of unrelated factors in the plasma samples. It is worth noting that there are few studies to evaluate the association between urinary biomarker and CAD. According to Sonmez’s report, 24-hour urinary albumin excretion rates may be used to determine patients with a high prospect of CAD [46]. Fitzsimmons et al. [47] also showed that urine MMP-9 and TIMP-1 levels were elevated in patients with CAD as compared with healthy volunteers. However, the discriminative powers of these CAD biomarkers were not as high as urinary CD14 shown in this report. In addition, urinary levels of CD14 are generally stable under experimental conditions, implying that urinary CD14 may possess greater potential as a favorable biomarker than other existing biomarkers for epidemiological and clinical application.

### Study limitations

Our study is limited by the fact that we cannot provide direct evidence for an increased recruitment of CD14+ monocytes into coronary atherosclerotic lesions. In addition, we cannot definitely show whether the increase in CD14+ monocytes precedes the development of atherosclerotic lesions, or whether it results from inflammatory conditions in atherosclerosis. Interpretation of the present results is limited by the small number of patients we studied. However, the correlations we found may serve as a reliable marker in the prediction and evaluation of CAD severity.

## Conclusions

The current study has demonstrated that release of CD14 in urine coupled with more CD14+ monocytes in CAD patients is significantly correlated with severity of CAD, pointing to the potential application of urinary CD14 as a novel biomarker for screening and diagnosis of patients manifested with different extents of CAD. Moreover, the relatively low abundance of urinary CD14 in subjects with normal coronary conditions may suggest the diagnostic and prognostic potentials to apply this CD14 as a sensitive biomarker for the detection of early cardiovascular disorders in selected cohort of populations.

## Supporting Information

S1 Materials and Methods (DOCX)

S1 Table Clinical characteristics in normal subjects and patients with coronary artery disease. (DOCX)

S2 Table Urinary soluble CD14 and associations with baseline characteristics of the study population (both patients and controls). (DOCX)

S3 Table Association of presence of CAD with CAD risk factors and urinary soluble CD14. (DOCX)

S4 Table Baseline characteristics and biochemical parameters of patients for flow cytometry. (DOCX)

S5 Table The quantitative shotgun analysis provided more than 100 identified proteins with different expression. (DOCX)
