# Supplementary material for: Clinical Proteomics Identifies Urinary CD14 as a Potential Biomarker for Diagnosis of Stable Coronary Artery Disease
Source: PLoS One. 2015 Feb 10;10(2):e0117169. doi: 10.1371/journal.pone.0117169 (PMC4323104; doi:10.1371/journal.pone.0117169)
Supplement: S1 Materials and Methods — (DOCX) [file pone.0117169.s001.docx]

**Materials and methods S1**

Chemicals and reagents

Quantitative reagents for protein contents were purchased from Bio-Rad (Hercules, CA). Trichloroacetic acid (TCA), triﬂuoroacetic acid (TFA), dithiothreitol (DTT), iodoacetamide (IAM), ethylenediaminetetraacetic acid (EDTA), sodium deoxycholate, sodium fluoride (NaF), formaldehyde-*H_2_*_,_ formaldehyde-*D_2_* and ammonium bicarbonate (NH_4_HCO_3_), Triton X-100 were purchased from Sigma Aldrich (St. Louis, MO). Acetonitrile (ACN) and sodium phosphate was obtained from Merck (Darmstadt, Germany). Formic acid (FA), sodium acetate, sodium cyanoborohydride and sodium chloride (NaCl) were purchased from Riedel-de Haven (Seelze, Germany). Proteinase inhibitors (Complete™ Mini) were purchased from Roche (Mannheim, Germany). Sodium dodecyl sulfate (SDS) and urea were purchased from Amresco (Solon, OH). Modiﬁed sequencing-grade trypsin for in-gel digestion was purchased from Promega (Madison, WI). Water was deionized to 18 MΩ by a Milli-Q system (Millipore, Bedford, MA).

Sample collection

All the procedures used in this study were approved by the ethical committee of clinical research at Kaohsiung Medical University Hospital. Urines from normal or CAD patients were lyophilized and subject to 1.5 mL extraction buffer (containing 10 mM Tris-HCl pH 7.4, 10 mM sodium phosphate, 150 mM NaCl, 0.1% SDS, 2 mM EDTA, 1% sodium deoxycholate, 100 mM NaF, 1% Triton X-100 and protease-inhibitor cocktail). The homogenates were transferred to 1.5 mL Eppendorf tubes and centrifuged at 13,000 x g for 20 min at 4°C to remove debris and insoluble material. Aliquots of the supernatants were assayed for determination of total protein concentration using Coomassie protein assay reagent, and subsequently were stored at -80°C until analyzed.

Dimethyl labeling and peptide preparation

100 μg of total urinal proteins from normal or CAD patients was adjusted to 60 μL and treated with 0.7 μL of 1 M DTT and 9.3 μL of 7.5% SDS at 95°C for 5 min before reduction. After the reaction, lysates were further treated with 8 μL of 50 mM IAM at room temperature for 30 min alkylation in the dark; subsequently proteins were precipitated by adding 52 μL of 50% TCA and incubated on ice for 15 min. After removing the supernatant by centrifugation at 13,000 x g for 5 min, the collected proteins were washed with 150 μL of 10% TCA, vortexed and centrifuged at 13,000 x g for 10 min. The precipitated proteins were washed again with 250 μL distilled H_2_O, vortexed and centrifuged under the same condition for 3 times. The resultant pellets were resuspended with 50 mM NH_4_HCO_3_ (pH 8.5), then digested with 4 μg of trypsin for 8 h at 37°C and further dried in a vacuum centrifuge to remove NH_4_HCO_3_. The lyophilized peptides for normal and CAD urine samples re-dissolved in 180 μL of 100 mM sodium acetate at pH 5.5 were treated with 20 μL of 4% formaldehyde-*H_2_* and 20 μL 4% formaldehyde-*D_2_*, respectively and mixed thoroughly.^1,2^ The mixtures were vortexed for 5 min, immediately followed by the addition of 20 μL of 3 M sodium cyanoborohydride and vortexed for 1 h at room temperature. The resultant liquids were acidified by 10% TFA/ H_2_O to pH 2.0~3.0 and applied onto the reverse phase C18 column pre-equilibrated with 200 μL of 0.1% TFA/H_2_O (pH 2.0~3.0). The column was also washed with 200 μL of 0.1% TFA/H_2_O (pH 3.0) and then eluted with a stepwise ACN gradient from 50% to 100% in 0.1% TFA at room temperature.

Hydrophilic interaction chromatography (HILIC) for peptide separation

HILIC was performed on an L-7100 pump system with quaternary gradient capability (Hitachi, Tokyo, Japan) using a TSK gel Amide-80 HILIC column (2.0 x 150 mm, 3 μm; Tosoh Biosciences, Tokyo, Japan) with a ﬂow rate of 200 μL/min.^3-5^ Two buﬀers were used for gradient elution: solvent (A), 0.1% TFA in 100% ACN, and solvent (B), 0.1% TFA in water. The eluted fractions from the reverse-phase C18 column were each dissolved in 50 μL of solution containing 85% ACN and 0.1% TFA and then injected into the 100 μL sample loop. The gradient was processed as follows: 0-2% (B) in 4 min, 2-8% (B) in 4 min, 8-12% (B) in 4 min, 12-35% (B) in 54 min, 35-100% (B) in 12 min and 100% (B) for 2 min. A total of 10 fractions were collected (1 mL for each fraction) and dried in a vacuum centrifuge.

Nano LC-MS/MS analysis

The lyophilized powders were reconstituted in 10 μL of 0.1% FA in H_2_O and analyzed by LTQ Orbitrap XL (Thermo Fisher Scientiﬁc, San Jose, CA). Reverse phase nano LC separation was performed on an Agilent 1200 series nanoﬂow system (Agilent Technologies, Santa Clara, CA). A total of 10 μL sample from collected fractions was loaded onto an Agilent Zorbax XDB C18 precolumn (0.35 mm, 5 μm), followed by separation using in-house handmade C18 column (i.d. 75 μm x 15-cm, 3 μm). The mobile phases used were (A) 0.1% FA and (B) 0.1% FA in 100% ACN. A linear gradient from 5% to 95% of (B) over a 70-min period at a ﬂow rate of 300 nL/min was applied. The peptides were analyzed in the positive ion mode by applying a voltage of 1.8 kV to the injection needle. The MS was operated in a data-dependent mode, in which one full scan with m/z 400-1600 in the Orbitrap using a scan rate of 30 ms/scan. The fragmentation was performed using the CID mode with a normalized collision energy of 35 V. A repeat duration of 30 s was applied to exclude the same m/z ions from the reselection for fragmentation. An in-house Xcalibur software (version 2.0.7, Thermo-Finnigan Inc., San Jose, CA) was used for the management of instrument control, data acquisition, and data processing.

Protein database search and characterization

Peptides were identiﬁed by peak lists converted from the nano LC-MS/MS spectra by bioinformatics searching against *Homo sapiens* taxonomy in the Swiss-Prot databases for exact matches using the Mascot search program (http://www.matrixscience. com).^6^ Parameters were set as follows: a mass tolerance of 10 ppm for precursor ions and 0.8 Da for fragment ions; no missed cleavage site allowed for trypsin; carbamidomethyl cysteine as fixed modification; dimethylation speciﬁed as the quantiﬁcation; oxidized methionine and amidated asparagine/glutamine as optional modification. Peptides were considered positively identiﬁed if their Mascot individual ion score was higher than 20 (p < 0.05). Subsequently, the analysis of peptide quantification ratio (D/H) for normal (hydrogen labeling) and CAD (deuterium labeling) was carried out by Mascot Distiller program (version 2.3, Matrix Science Ltd., London, U.K.) using the average area of the first 3 isotopic peaks across the elution profile. The Mascot search data as well as quantiﬁcation results from each fraction were also merged by this program that combined the peptide ratios matching the same sequence obtained from different fractions or at different retention time and charge state.

Construction of signaling pathways and network analysis of protein interaction

The software program (www.ingenuity.com) from Ingenuity Pathways Analysis (IPA, Ingenuity Systems, Redwood City, CA) was used for deriving the pathways and networks of protein interaction, and the involved prospective mechanism. Protein factors characterized by proteomic analysis were analyzed for their association with mapping related to canonical pathways deposited in the IPA library.

**Supplemental References**

1. Boersema PJ, Aye TT, van Veen TA, Heck AJ, Mohammed S (2008) Triplex protein quantification based on stable isotope labeling by peptide dimethylation applied to cell and tissue lysates. Proteomics 8: 4624-4632.

2. Yoshida T (1997) Peptide separation in normal phase liquid chromatography. Anal Chem 69: 3038-3043.

3. Yoshida T (1998) Calculation of peptide retention coefficients in normal-phase liquid chromatography. J Chromatogr A 808: 105-112.

4. Alpert AJ (1990) Hydrophilic-interaction chromatography for the separation of peptides, nucleic acids and other polar compounds. J Chromatogr 499: 177-196.

5. Perkins DN, Pappin DJ, Creasy DM, Cottrell JS (1999) Probability-based protein identification by searching sequence databases using mass spectrometry data. Electrophoresis 20: 3551-3567.

6. Hirosawa M, Hoshida M, Ishikawa M, Toya T (1993) MASCOT: multiple alignment system for protein sequences based on three-way dynamic programming. Comput Appl Biosci 9: 161-167.
