# Supplementary material for: Clinical Proteomics Identifies Urinary CD14 as a Potential Biomarker for Diagnosis of Stable Coronary Artery Disease
Source: PLoS One. 2015 Feb 10;10(2):e0117169. doi: 10.1371/journal.pone.0117169 (PMC4323104; doi:10.1371/journal.pone.0117169)
Supplement: S1 Table — (DOCX) [file pone.0117169.s002.docx]

**Table S1.** Clinical characteristics in normal subjects and patients with coronary artery disease.

|  | **Normal**  **(n＝35)** | **Single vessel CAD**  **(n＝20)** | **Multi-vessel CAD**  **(n＝53)** | ***P* value** |
| --- | --- | --- | --- | --- |
| Age (yrs) | 61.1±13.4 | 64.0±12.2 | 68.9±11.1 | 0.011 |
| Gender (F/M) | 19/16 | 7/13 | 13/40 | 0.029 |
| Body mass index (kg/m^2^) | 27.9±5.0 | 27.0±2.7 | 25.7±3.6 | 0.689 |
| Diabetes (%) | 8.6 | 35.0 | 36.0 | 0.011 |
| Hypertension (%) | 77.0 | 80.2 | 81.4 | 0.812 |
| Smoking status (%) |  |  |  |  |
| Current smokers | 27.0 | 26.0 | 31.0 | 0.347 |
| Ex-smokers | 31.0 | 32.0 | 29.0 | 0.871 |
| Non-smokers | 42.0 | 42.0 | 40.0 | 0.901 |
| Systolic blood pressure (mmHg) | 177.8±31.1 | 177.4±27.8 | 180.5±31.0 | 0.910 |
| Diastolic blood pressure (mmHg) | 81.7±13.7 | 81.5±10.9 | 78.9±11.7 | 0.782 |
| LVEF (%) | 71.8±8.1 | 65.4±15.2 | 64.3±15.4 | 0.688 |
| Serum creatinine (mg/dL) | 0.89±0.25 | 0.94±0.27 | 1.05±0.29 | 0.039 |
| Total cholesterol (mg/dL) | 185.9±41.9 | 166.2±31.6 | 188.4±66.3 | 0.452 |
| LDL cholesterol (mg/dL) | 114.6±32.9 | 102.8±31.2 | 119.3±49.4 | 0.671 |
| HDL cholesterol (mg/dL) | 43.8±13.5 | 43.7±10.8 | 40.7±9.9 | 0.712 |
| Triglycerides (mg/dL) | 136.9±66.4 | 113.7±52.9 | 166.8±153.0 | 0.321 |
| Hemoglobin A_1C_ (%) | 5.7 | 6.2 | 6.8 | 0.043 |
| hs-CRP (mg/dL) | 0.64 (0.33-0.75) | 0.35 (0.25-0.42) | 0.86 (0.36-1.08) | 0.882 |
| Fibrinogen (mg/dL) | 438.1±107.2 | 476.2±120.9 | 452.6±80.9 | 0.485 |
| Total white cells (10^3^/ml) | 7036±1974 | 7048±1710 | 8141±3953 | 0.129 |
| Neutrophils (%) | 60.6 | 61.5 | 63.5 | 0.719 |
| Lymphocytes (%) | 31.1 | 29.0 | 27.5 | 0.141 |
| Monocytes (%) | 5.3 | 6.4 | 5.9 | 0.192 |
| Eosinophils (%) | 2.2 | 2.6 | 2.6 | 0.227 |
| Basophils (%) | 0.5 | 0.4 | 0.4 | 0.340 |
| Urinary sCD14 (ug/mL) | 2.08 (1.38-2.77) | 9.55 (3.76-14.10) | 11.10 (8.08-15.33) | <0.001 |
| Serum sCD14 (ug/mL) | 125.88±35.85 | 96.07±24.67 | 130.01±32.81 | 0.089 |
| log Urinary sCD14 | 0.29±0.23 | 0.75±0.41 | 0.93±0.32 | <0.001 |
| Urinary protein (mg/dL) | 34.5±115.1 | 22.6±48.6 | 24.1±46.4 | 0.081 |
| Urinary microalbumin (mg/L) | 231.0±810.6 | 106.7±233.5 | 101.9±304.8 | 0.059 |
| UACR | 1.66±4.43 | 1.30±3.10 | 1.76±5.64 | 0.239 |
| SYNTAX score | 0.0 (0.0-0.0) | 7.3 (5.7-9.0) | 23.0 (19.0-24.7) | <0.001 |
| Antiplatelet drugs (%) | 83.0 | 90.0 | 94.0 | 0.329 |
| Diuretics (%) | 11.0 | 25.0 | 21.0 | 0.238 |
| Beta-blockers (%) | 37.0 | 35.0 | 45.0 | 0.301 |
| ACE inhibitors (%) | 40.0 | 35.0 | 36.0 | 0.219 |
| ARBs (%) | 11.0 | 15.0 | 13.0 | 0.302 |
| Fibrates (%) | 2.9 | 7.6 | 4.6 | 0.118 |
| Statins (%) | 2.9 | 5.0 | 8.3 | 0.110 |

Data are presented as the mean value ± SD. The hs-CRP and urinary CD14 levels and SYNTAX score are presented as the median and interquartile range.

CAD, coronary artery disease; NS, not significant; LVEF, left ventricular ejection fraction; LDL cholesterol, low density lipoprotein cholesterol; HDL cholesterol, high density lipoprotein cholesterol; hs-CRP, high sensitivity C-reactive protein; UACR, urinary microalbumin/creatinine ratio; ACE, angiotensin-converting enzyme; ARB, angiotensinogen receptor blocker.
