# Supplementary material for: Clinical Proteomics Identifies Urinary CD14 as a Potential Biomarker for Diagnosis of Stable Coronary Artery Disease
Source: PLoS One. 2015 Feb 10;10(2):e0117169. doi: 10.1371/journal.pone.0117169 (PMC4323104; doi:10.1371/journal.pone.0117169)
Supplement: S2 Table — (DOCX) [file pone.0117169.s003.docx]

**Table S2.** Urinary CD14 and associations with baseline characteristics of the study population (both patients and controls).

|  | **Urinary CD14 quartiles** | | | | | |
| --- | --- | --- | --- | --- | --- | --- |
| **Both sexes** | **1**  **(0-1.91 ug/mL)** | | **2**  **(2.00-4.51 ug/mL)** | **3**  **(4.60-9.09 ug/mL)** | **4**  **(9.10-50.30 ug/mL)** | ***P* value** |
| Age (yrs) | 60.9±14.3 | 64.0±11.3 | | 63.4±9.7 | 73.7±10.7 | 0.001 |
| Body mass index (kg/m^2^) | 27.9±4.7 | 26.7±4.3 | | 27.0±4.1 | 25.0±3.0 | 0.761 |
| Diabetes (%) | 11.0 | 37.0 | | 26.0 | 33.0 | 0.162 |
| Hypertension (%) | 70.0 | 85.0 | | 78.0 | 85.0 | 0.720 |
| Smoking status, current (%) | 25.0 | 33.0 | | 27.0 | 26.0 | 0.239 |
| Serum creatinine (mg/dL) | 0.86±0.23 | 0.98±0.28 | | 1.02±0.29 | 1.04±0.30 | 0.642 |
| Total cholesterol (mg/dL) | 198.7±91.1 | 184.0±28.5 | | 180.9±30.6 | 169.9±40.3 | 0.810 |
| LDL cholesterol (mg/dL) | 124.8±63.2 | 111.8±26.9 | | 108.2±29.0 | 114.5±37.4 | 0.792 |
| HDL cholesterol (mg/dL) | 45.6±15.1 | 42.8±9.5 | | 41.4±10.4 | 39.2±9.1 | 0.808 |
| Triglycerides (mg/dL) | 154.3±125.5 | 145.7±95.1 | | 182.1±161.8 | 104.6±41.0 | 0.217 |
| hs-CRP (mg/dL) | 0.66 (0.27-0.77) | 0.61 (0.36-2.70) | | 0.47 (0.24-0.85) | 0.71 (0.37-1.91) | 0.159 |
| Fibrinogen (mg/dL) | 429.3±103.7 | 444.3±56.3 | | 471.7±123.6 | 471.6±110.9 | 0.239 |
| SYNTAX score | 0.0 (0.0-0.0) | 11.5 (5.3-13.3) | | 24.0 (13.0-29.0) | 24.0 (19.0-32.0) | <0.001 |
| Serum CD14 (ug/mL) | 126.3±40.9 | 119.7±28.3 | | 107.1±38.5 | 135.8±32.7 | 0.094 |

Data are presented as the mean value ± SD. The hs-CRP and SYNTAX score are presented as the median and interquartile range.

NS, not significant; LDL cholesterol, low density lipoprotein cholesterol; HDL cholesterol, high density lipoprotein cholesterol; hs-CRP, high sensitivity C-reactive protei
