# Supplementary material for: Clinical Proteomics Identifies Urinary CD14 as a Potential Biomarker for Diagnosis of Stable Coronary Artery Disease
Source: PLoS One. 2015 Feb 10;10(2):e0117169. doi: 10.1371/journal.pone.0117169 (PMC4323104; doi:10.1371/journal.pone.0117169)
Supplement: S3 Table — (DOCX) [file pone.0117169.s004.docx]

**Table S3.** Association of presence of CAD with CAD risk factors and urinary CD14.

| **Variable** | **OR** | **95% CI** | ***P* value** |
| --- | --- | --- | --- |
| Age | 1.008 | 0.932–1.091 | 0.834 |
| Male gender | 1.537 | 0.233–4.985 | 0.367 |
| Body mass index | 1.437 | 0.257–2.054 | 0.652 |
| Diabetes | 3.013 | 1.933–4.692 | 0.027 |
| Hypertension | 1.134 | 0.013–6.114 | 0.294 |
| Current smoking | 2.943 | 0.359–4.840 | 0.033 |
| Hypercholesterolemia | 0.982 | 0.953–1.011 | 0.156 |
| Serum creatinine | 2.772 | 0.299–5.724 | 0.369 |
| hs-CRP | 0.744 | 0.412–1.339 | 0.322 |
| Urinary CD14 | 3.336 | 1.232–9.032 | 0.018 |

| hs-CRP, high sensitivity C-reactive protein. |
| --- |
