# Supplementary material for: Clinical Proteomics Identifies Urinary CD14 as a Potential Biomarker for Diagnosis of Stable Coronary Artery Disease
Source: PLoS One. 2015 Feb 10;10(2):e0117169. doi: 10.1371/journal.pone.0117169 (PMC4323104; doi:10.1371/journal.pone.0117169)
Supplement: S4 Table — (DOCX) [file pone.0117169.s005.docx]

**Table S4.** Baseline characteristics and biochemical parameters of patients for flow cytometry.

|  | **Patients without CAD**  **(n＝5)** | **Patients with CAD**  **(n＝5)** |
| --- | --- | --- |
| Age, years | 67.2±11.8 | 68.4±10.8 |
| Gender (male, %) | 60 | 60 |
| BMI, kg/m^2^ | 25.5±4.8 | 25.9±3.5 |
| Diabetes, % | 40 | 40 |
| Hypertension, % | 80 | 80 |
| Current smoking, % | 40 | 40 |
| Systolic blood pressure, mmHg | 178±31 | 179±30 |
| Diastolic blood pressure, mmHg | 77±12 | 78±12 |
| Leukocyte count, cells/mm^3^ | 6380±2030 | 8410±3390 |
| Fasting glucose, mg/dL | 120.8±66.2 | 122.2±65.8 |
| Hemoglobin A_1_c, % | 6.5±1.8 | 6.9±1.9 |
| Uric acid, mg/dL | 6.2±1.5 | 6.2±1.8 |
| Total cholesterol, mg/dL | 190.0±49.9 | 196.9±42.6 |
| LDL cholesterol, mg/dL | 117.2±29.9 | 120.6±40.8 |
| HDL cholesterol, mg/dL | 45.4±12.6 | 45.4±13.3 |
| Triglycerides, mg/dL | 149.3±130.5 | 138.8±76.7 |
| Serum creatinine, mg/dL | 1.00±0.32 | 1.02±0.31 |
| hs-CRP, mg/dl | 1.49±2.52 | 1.87±7.95 |
| LVEF, % | 68.4±11.7 | 66.3±12.5 |
| SYNTAX score | 0 | 15.4 |
| Medications |  |  |
| Aspirin, % | 86 | 89 |
| Calcium antagonists, % | 31 | 28 |
| Beta-blockers, % | 52 | 38 |
| Angiotensin-converting enzyme inhibitors, % | 21 | 38 |
| Angiotensin II receptor blockers, % | 21 | 17 |
| Loop diuretics, % | 7 | 13 |
| Thiazides, % | 7 | 2 |
| Spironolactone, % | 7 | 13 |
| Statins, % | 9 | 11 |
| Fibrates, % | 5 | 8 |

Data are expressed as mean + SD.

BMI, body mass index; LDL, low density lipoprotein; HDL, high density lipoprotein; hs-CRP, high sensitivity C-reactive protein; LVEF, left ventricular ejection fraction.
