# Supplementary material for: Clinical Proteomics Identifies Urinary CD14 as a Potential Biomarker for Diagnosis of Stable Coronary Artery Disease
Source: PLoS One. 2015 Feb 10;10(2):e0117169. doi: 10.1371/journal.pone.0117169 (PMC4323104; doi:10.1371/journal.pone.0117169)
Supplement: S5 Table — (DOCX) [file pone.0117169.s006.docx]

**Table S5.** The quantitative shotgun analysis provided more than 100 identified proteins with different expression.

| Hit | Accession | Score | Mass | H/L | SD(geo) | # | Description |
| --- | --- | --- | --- | --- | --- | --- | --- |
| 7 | IGHG2 | 322 | 37178 |  |  |  | Ig gamma-2 chain C region OS=Homo sapiens GN=IGHG2 PE=1 SV=2 |
| 80 | AMNLS | 26 | 48594 | 812.9 | 3.777 | 2 | Protein amnionless OS=Homo sapiens GN=AMN PE=1 SV=2 |
| 75 | DYRK3 | 27 | 68009 | 300.6 | 11.05 | 2 | Dual specificity tyrosine-phosphorylation-regulated kinase 3 OS=Homo sapiens GN=DYRK3 PE=1 SV=3 |
| 70 | HPHL1 | 31 | 134757 | 38.74 | 1.769 | 2 | Hephaestin-like protein 1 OS=Homo sapiens GN=HEPHL1 PE=2 SV=2 |
| 47 | AMPE | 54 | 111174 | 25.88 | 83.23 | 2 | Glutamyl aminopeptidase OS=Homo sapiens GN=ENPEP PE=1 SV=3 |
| 89 | CR001 | 23 | 34698 | 25.19 | 1.066 | 2 | Uncharacterized protein C18orf1 OS=Homo sapiens GN=C18orf1 PE=2 SV=1 |
| 62 | XPO2 | 34 | 112770 | 15.59 | 1.332 | 3 | Exportin-2 OS=Homo sapiens GN=CSE1L PE=1 SV=3 |
| 9 | CD44 | 300 | 82642 | 14 | 2.411 | 6 | CD44 antigen OS=Homo sapiens GN=CD44 PE=1 SV=3 |
| 12 | VASN | 235 | 73007 | 10.68 | 1.826 | 4 | Vasorin OS=Homo sapiens GN=VASN PE=1 SV=1 |
| 21 | BGAL | 134 | 77444 | 9.89 | 1.727 | 2 | Beta-galactosidase OS=Homo sapiens GN=GLB1 PE=1 SV=2 |
| 93 | CD14 | 23 | 40966 | 9.682 | 1.063 | 2 | Monocyte differentiation antigen CD14 OS=Homo sapiens GN=CD14 PE=1 SV=2 |
| 58 | AMYP | 38 | 59092 | 9.664 | 1.438 | 2 | Pancreatic alpha-amylase OS=Homo sapiens GN=AMY2A PE=1 SV=2 |
| 23 | GGH | 129 | 37078 | 8.287 | 2.032 | 2 | Gamma-glutamyl hydrolase OS=Homo sapiens GN=GGH PE=1 SV=2 |
| 56 | ASAH1 | 41 | 45920 | 6.5 | 1.165 | 2 | Acid ceramidase OS=Homo sapiens GN=ASAH1 PE=1 SV=5 |
| 16 | THRB | 181 | 72404 | 5.984 | 2.101 | 9 | Prothrombin OS=Homo sapiens GN=F2 PE=1 SV=2 |
| 20 | CADH1 | 145 | 98833 | 5.4 | 1.286 | 2 | Cadherin-1 OS=Homo sapiens GN=CDH1 PE=1 SV=3 |
| 14 | KNG1 | 200 | 74630 | 4.574 | 3.124 | 7 | Kininogen-1 OS=Homo sapiens GN=KNG1 PE=1 SV=2 |
| 53 | YIPF3 | 42 | 38707 | 4.556 | 1.03 | 2 | Protein YIPF3 OS=Homo sapiens GN=YIPF3 PE=1 SV=1 |
| 15 | PTGDS | 200 | 21564 | 4.405 | 4.053 | 3 | Prostaglandin-H2 D-isomerase OS=Homo sapiens GN=PTGDS PE=1 SV=1 |
| 39 | SULF2 | 65 | 103939 | 4.298 | 1.021 | 2 | Extracellular sulfatase Sulf-2 OS=Homo sapiens GN=SULF2 PE=1 SV=1 |
| 8 | PIGR | 310 | 85606 | 4.09 | 3.592 | 12 | Polymeric immunoglobulin receptor OS=Homo sapiens GN=PIGR PE=1 SV=4 |
| 51 | IBP7 | 46 | 30558 | 3.925 | 1.142 | 2 | Insulin-like growth factor-binding protein 7 OS=Homo sapiens GN=IGFBP7 PE=1 SV=1 |
| 61 | IGHA1 | 34 | 38903 | 3.854 | 1.367 | 3 | Ig alpha-1 chain C region OS=Homo sapiens GN=IGHA1 PE=1 SV=2 |
| 44 | LRP2 | 58 | 544973 | 3.486 | 1.209 | 2 | Low-density lipoprotein receptor-related protein 2 OS=Homo sapiens GN=LRP2 PE=1 SV=3 |
| 71 | FUCO | 30 | 54741 | 3.431 | 1.267 | 2 | Tissue alpha-L-fucosidase OS=Homo sapiens GN=FUCA1 PE=1 SV=4 |
| 30 | DNAS1 | 88 | 31866 | 3.291 | 3.346 | 5 | Deoxyribonuclease-1 OS=Homo sapiens GN=DNASE1 PE=1 SV=1 |
| 24 | HEG1 | 119 | 150144 | 2.756 | 1.002 | 2 | Protein HEG homolog 1 OS=Homo sapiens GN=HEG1 PE=1 SV=3 |
| 32 | RNAS1 | 87 | 18377 | 2.716 | 4.013 | 5 | Ribonuclease pancreatic OS=Homo sapiens GN=RNASE1 PE=1 SV=4 |
| 2 | AMBP | 7074 | 40463 | 2.701 | 2.33 | 108 | Protein AMBP OS=Homo sapiens GN=AMBP PE=1 SV=1 |
| 1 | UROM | 28757 | 72964 | 2.641 | 4.069 | 416 | Uromodulin OS=Homo sapiens GN=UMOD PE=1 SV=1 |
| 38 | CD59 | 67 | 14991 | 2.534 | 1.074 | 3 | CD59 glycoprotein OS=Homo sapiens GN=CD59 PE=1 SV=1 |
| 49 | LG3BP | 47 | 66811 | 2.176 | 1.014 | 2 | Galectin-3-binding protein OS=Homo sapiens GN=LGALS3BP PE=1 SV=1 |
| 52 | IC1 | 44 | 56277 | 2.171 | 1.716 | 2 | Plasma protease C1 inhibitor OS=Homo sapiens GN=SERPING1 PE=1 SV=2 |
| 35 | ZG16B | 71 | 23005 | 1.776 | 1.01 | 3 | Zymogen granule protein 16 homolog B OS=Homo sapiens GN=ZG16B PE=1 SV=3 |
| 10 | LAMP2 | 254 | 46080 | 1.653 | 1.001 | 2 | Lysosome-associated membrane glycoprotein 2 OS=Homo sapiens GN=LAMP2 PE=1 SV=2 |
| 27 | FETUA | 96 | 40611 | 1.64 | 1.229 | 4 | Alpha-2-HS-glycoprotein OS=Homo sapiens GN=AHSG PE=1 SV=1 |
| 69 | A1AT | 31 | 47831 | 1.41 | 47.34 | 2 | Alpha-1-antitrypsin OS=Homo sapiens GN=SERPINA1 PE=1 SV=3 |
| 25 | LAMP1 | 112 | 45872 | 1.389 | 1.069 | 6 | Lysosome-associated membrane glycoprotein 1 OS=Homo sapiens GN=LAMP1 PE=1 SV=3 |
| 17 | KV302 | 172 | 11978 | 1.337 | 2.382 | 7 | Ig kappa chain V-III region SIE OS=Homo sapiens PE=1 SV=1 |
| 4 | IGKC | 1022 | 11997 | 1.221 | 1.241 | 18 | Ig kappa chain C region OS=Homo sapiens GN=IGKC PE=1 SV=1 |
| 41 | MGA | 62 | 213083 | 1.094 | 1.997 | 4 | Maltase-glucoamylase, intestinal OS=Homo sapiens GN=MGAM PE=1 SV=5 |
| 19 | LYVE1 | 150 | 36462 | 1.044 | 1.126 | 4 | Lymphatic vessel endothelial hyaluronic acid receptor 1 OS=Homo sapiens GN=LYVE1 PE=1 SV=2 |
| 46 | PGCP | 55 | 52852 | 1.01 | 6.762 | 3 | Plasma glutamate carboxypeptidase OS=Homo sapiens GN=PGCP PE=1 SV=1 |
| 13 | SAP3 | 215 | 21589 | 0.9701 | 1.323 | 7 | Ganglioside GM2 activator OS=Homo sapiens GN=GM2A PE=1 SV=4 |
| 22 | LAC2 | 134 | 11682 | 0.8309 | 1.095 | 7 | Ig lambda-2 chain C regions OS=Homo sapiens GN=IGLC2 PE=1 SV=1 |
| 18 | APOH | 172 | 40425 | 0.6091 | 1.179 | 2 | Beta-2-glycoprotein 1 OS=Homo sapiens GN=APOH PE=1 SV=3 |
| 50 | VTDB | 47 | 55759 | 0.5205 | 1.064 | 2 | Vitamin D-binding protein OS=Homo sapiens GN=GC PE=1 SV=1 |
| 11 | ZA2G | 244 | 35026 | 0.4838 | 2.654 | 9 | Zinc-alpha-2-glycoprotein OS=Homo sapiens GN=AZGP1 PE=1 SV=2 |
| 31 | TRFE | 88 | 80920 | 0.399 | 2.535 | 4 | Serotransferrin OS=Homo sapiens GN=TF PE=1 SV=3 |
| 6 | OSTP | 464 | 36105 | 0.3519 | 2.593 | 14 | Osteopontin OS=Homo sapiens GN=SPP1 PE=1 SV=1 |
| 3 | ALBU | 3683 | 72999 | 0.2841 | 2.309 | 125 | Serum albumin OS=Homo sapiens GN=ALB PE=1 SV=2 |
| 29 | A2GL | 89 | 38747 | 0.227 | 1.109 | 5 | Leucine-rich alpha-2-glycoprotein OS=Homo sapiens GN=LRG1 PE=1 SV=2 |
| 5 | A1AG1 | 740 | 24117 | 0.2057 | 2.261 | 22 | Alpha-1-acid glycoprotein 1 OS=Homo sapiens GN=ORM1 PE=1 SV=1 |
| 5 | A1AG2 | 316 | 24237 | 0.1866 | 2.307 | 7 | Alpha-1-acid glycoprotein 2 OS=Homo sapiens GN=ORM2 PE=1 SV=2 |
| 48 | PSCA | 53 | 13614 | 0.00521 | 1.593 | 2 | Prostate stem cell antigen OS=Homo sapiens GN=PSCA PE=1 SV=1 |
| 77 | DUOX1 | 26 | 180009 | 0.00186 | 11.31 | 3 | Dual oxidase 1 OS=Homo sapiens GN=DUOX1 PE=1 SV=1 |
| 26 | DPP4 | 111 | 90189 |  |  |  | Dipeptidyl peptidase 4 OS=Homo sapiens GN=DPP4 PE=1 SV=2 |
| 28 | ARSA | 92 | 54665 |  |  |  | Arylsulfatase A OS=Homo sapiens GN=ARSA PE=1 SV=3 |
| 33 | ANAG | 84 | 83054 |  |  |  | Alpha-N-acetylglucosaminidase OS=Homo sapiens GN=NAGLU PE=1 SV=2 |
| 34 | LYAG | 76 | 106533 |  |  |  | Lysosomal alpha-glucosidase OS=Homo sapiens GN=GAA PE=1 SV=4 |
| 36 | TSN1 | 69 | 27247 |  |  |  | Tetraspanin-1 OS=Homo sapiens GN=TSPAN1 PE=1 SV=2 |
| 37 | P3IP1 | 69 | 28975 |  |  |  | Phosphoinositide-3-kinase-interacting protein 1 OS=Homo sapiens GN=PIK3IP1 PE=1 SV=2 |
| 40 | PGBM | 65 | 481159 |  |  |  | Basement membrane-specific heparan sulfate proteoglycan core protein OS=Homo sapiens GN=HSPG2  PE=1 SV=4 |
| 42 | APOD | 61 | 21855 |  |  |  | Apolipoprotein D OS=Homo sapiens GN=APOD PE=1 SV=1 |
| 43 | CETP | 60 | 55954 |  |  |  | Cholesteryl ester transfer protein OS=Homo sapiens GN=CETP PE=1 SV=2 |
| 45 | PPAL | 57 | 49065 |  |  |  | Lysosomal acid phosphatase OS=Homo sapiens GN=ACP2 PE=1 SV=3 |
| 54 | KV401 | 42 | 13598 |  |  |  | Ig kappa chain V-IV region (Fragment) OS=Homo sapiens GN=IGKV4-1 PE=4 SV=1 |
| 55 | NUD22 | 41 | 32973 |  |  |  | Nucleoside diphosphate-linked moiety X motif 22 OS=Homo sapiens GN=NUDT22 PE=2 SV=3 |
| 57 | ARSB | 40 | 60900 |  |  |  | Arylsulfatase B OS=Homo sapiens GN=ARSB PE=1 SV=1 |
| 59 | CA190 | 35 | 26217 |  |  |  | NF-kappa-B activator C1orf190 OS=Homo sapiens GN=C1orf190 PE=1 SV=1 |
| 60 | ACTA | 35 | 42914 |  |  |  | Actin, aortic smooth muscle OS=Homo sapiens GN=ACTA2 PE=1 SV=1 |
| 63 | PEPA | 33 | 42606 |  |  |  | Pepsin A OS=Homo sapiens GN=PGA3 PE=1 SV=1 |
| 64 | YAP1 | 32 | 54877 |  |  |  | Yorkie homolog OS=Homo sapiens GN=YAP1 PE=1 SV=2 |
| 65 | MPRI | 32 | 285739 |  |  |  | Cation-independent mannose-6-phosphate receptor OS=Homo sapiens GN=IGF2R PE=1 SV=3 |
| 66 | RGAG1 | 31 | 146021 |  |  |  | Retrotransposon gag domain-containing protein 1 OS=Homo sapiens GN=RGAG1 PE=1 SV=1 |
| 67 | KI3L3 | 31 | 45722 |  |  |  | Killer cell immunoglobulin-like receptor 3DL3 OS=Homo sapiens GN=KIR3DL3 PE=2 SV=2 |
| 68 | S23IP | 31 | 113289 |  |  |  | SEC23-interacting protein OS=Homo sapiens GN=SEC23IP PE=1 SV=1 |
| 72 | RPB1 | 29 | 221351 |  |  |  | DNA-directed RNA polymerase II subunit RPB1 OS=Homo sapiens GN=POLR2A PE=1 SV=2 |
| 73 | SZT2 | 29 | 383969 |  |  |  | Protein SZT2 OS=Homo sapiens GN=SZT2 PE=2 SV=3 |
| 74 | GOGB1 | 28 | 386017 |  |  |  | Golgin subfamily B member 1 OS=Homo sapiens GN=GOLGB1 PE=1 SV=2 |
| 76 | NHLC1 | 27 | 43684 |  |  |  | E3 ubiquitin-protein ligase NHLRC1 OS=Homo sapiens GN=NHLRC1 PE=1 SV=2 |
| 78 | ANR42 | 26 | 44239 |  |  |  | Ankyrin repeat domain-containing protein 42 OS=Homo sapiens GN=ANKRD42 PE=2 SV=2 |
| 79 | ODO2 | 26 | 49823 |  |  |  | Dihydrolipoyllysine-residue succinyltransferase component of 2-oxoglutarate dehydrogenase complex,  mitochondrial OS=Homo sapiens GN=DLST PE=1 SV=4 |
| 81 | GCP3 | 25 | 105619 |  |  |  | Gamma-tubulin complex component 3 OS=Homo sapiens GN=TUBGCP3 PE=1 SV=2 |
| 82 | EPCR | 25 | 27061 |  |  |  | Endothelial protein C receptor OS=Homo sapiens GN=PROCR PE=1 SV=1 |
| 83 | KYNU | 25 | 54050 |  |  |  | Kynureninase OS=Homo sapiens GN=KYNU PE=1 SV=1 |
| 84 | A1BG | 25 | 55098 |  |  |  | Alpha-1B-glycoprotein OS=Homo sapiens GN=A1BG PE=1 SV=4 |
| 85 | MACC1 | 25 | 99170 |  |  |  | Metastasis-associated in colon cancer protein 1 OS=Homo sapiens GN=MACC1 PE=1 SV=2 |
| 86 | THBG | 24 | 47422 |  |  |  | Thyroxine-binding globulin OS=Homo sapiens GN=SERPINA7 PE=1 SV=2 |
| 87 | SIX3 | 24 | 36199 |  |  |  | Homeobox protein SIX3 OS=Homo sapiens GN=SIX3 PE=1 SV=1 |
| 88 | CSTF2 | 24 | 61451 |  |  |  | Cleavage stimulation factor subunit 2 OS=Homo sapiens GN=CSTF2 PE=1 SV=1 |
| 90 | GSHB | 23 | 53229 |  |  |  | Glutathione synthetase OS=Homo sapiens GN=GSS PE=1 SV=1 |
| 91 | CLC4G | 23 | 33457 |  |  |  | C-type lectin domain family 4 member G OS=Homo sapiens GN=CLEC4G PE=1 SV=1 |
| 92 | K1549 | 23 | 212887 |  |  |  | UPF0606 protein KIAA1549 OS=Homo sapiens GN=KIAA1549 PE=1 SV=4 |
| 94 | LMBL2 | 23 | 81849 |  |  |  | Lethal(3)malignant brain tumor-like protein 2 OS=Homo sapiens GN=L3MBTL2 PE=1 SV=1 |
| 95 | PROM1 | 22 | 99684 |  |  |  | Prominin-1 OS=Homo sapiens GN=PROM1 PE=1 SV=1 |
| 96 | PKNX2 | 22 | 52890 |  |  |  | Homeobox protein PKNOX2 OS=Homo sapiens GN=PKNOX2 PE=1 SV=2 |
| 97 | ELF1 | 21 | 68777 |  |  |  | ETS-related transcription factor Elf-1 OS=Homo sapiens GN=ELF1 PE=1 SV=2 |
| 98 | HNRPM | 21 | 79032 |  |  |  | Heterogeneous nuclear ribonucleoprotein M OS=Homo sapiens GN=HNRNPM PE=1 SV=3 |
